# Dr. J. B. Hamilton

**Published:** 1899-01

**Authors:** 


### OBITUARY.

### Dr. J. B. Hamilton.

Died, December 24, 1898, at his residence, Elgin, Ills., Dr. J. B. Hamilton, editor of the Journal of the American Medical Association, aged fifty-one years.

Dr. Hamilton has had a remarkable career, one that has brought him prominently before the medical profession of this country, and indeed to the attention of the civilized world.

At the early age of seventeen years he entered the Union Army in the Civil War, with all the enthusiasm of a young, vigorous and aspiring life. At the close of the war he entered Rush Medical College of Chicago, and graduated three years later with high honors. He began the practice of medicine in his native place, Jersey County, Ills. He was not long content, however, in a position of this kind, he entered the career of a military surgeon and returned to the army of the United States as assistant surgeon. He was rapidly transferred from one position to another, and in positions of responsibility he gave evidence of rare ability for the organization and direction of great enterprises, indeed this seems to be a feature of his character. In 1876 he transferred his connection to the United States Marine Hospital Service, in this his superior powers asserted themselves. He rapidly rose from one position to another, till within three or four years he had reached that of Surgeon General of the United States Marine Hospital Service. This he retained through several administrations of the National Government, performing acceptably every duty assigned him. In the halls of legislation his influence was as beneficently potent as in the field of medical action. It is no exaggeration to claim that the most important acts for the security of the public health by the National Legislature during the past twenty years were originated and guided by the enlightened intellect of Dr. Hamilton. He has been Professor of Surgery in several medical schools, indeed at the time of his death he was Professor in Rush Medical College. He was for many years an active member of the American Medical Association. He exercised a powerful influence in the sanitary movements of our country. Whenever there was a work in operation pertaining to medicine or sanitation Dr. Hamilton's influence, either personally or otherwise, was sure to be manifest. He was Secretary of the Seventh International Medical Congress, held at Washington, D.C., in 1887; he was an efficient member of its executive commitee. This action gave him a great amount of intensely laborious work, all of which he discharged to the entire satisfaction of all interested. We are induced to speak thus of Dr. Hamilton from a long acquaintance and a knowledge of the great work he did. He was one of the warmest friends and active supporters of the Dental Section of the Seventh International Medical Congress, and with one exception, possibly, did more than any other medical man to promote its interest and make it the success it was.

In the death of Dr. Hamilton our profession of this country has lost an appreciative friend, and one who was ever ready to give his work and his influence for its interest.

